# Pulmonary Embolism in Critically Ill Patients—Prevention, Diagnosis, and Management

**DOI:** 10.3390/diagnostics14192208

**Published:** 2024-10-03

**Authors:** Charikleia S. Vrettou, Effrosyni Dima, Ioanna Sigala

**Affiliations:** First Department of Critical Care Medicine, Evangelismos Hospital, Medical School, National & Kapodistrian University of Athens, 10676 Athens, Greecegiannasig@yahoo.com (I.S.)

**Keywords:** thromboembolic disease, pulmonary embolism, intensive care unit

## Abstract

Critically ill patients in the intensive care unit (ICU) are often immobilized and on mechanical ventilation, placing them at increased risk for thromboembolic diseases, particularly deep vein thrombosis (DVT) and, to a lesser extent, pulmonary embolism (PE). While these conditions are frequently encountered in the emergency department, managing them in the ICU presents unique challenges. Although existing guidelines are comprehensive and effective, they are primarily designed for patients presenting with PE in the emergency department and do not fully address the complexities of managing critically ill patients in the ICU. This review aims to summarize the available data on these challenging cases, offering a practical approach to the prevention, diagnosis, and treatment of PE, particularly when it is acquired in the ICU.

## 1. Introduction

Pulmonary embolism (PE) is recognized as a significant preventable cause of hospital morbidity and mortality. Critically ill patients treated in intensive care units (ICUs) are at high risk for thromboembolic disease, both deep vein thrombosis (DVT) and PE [1]. Key factors contributing to this elevated risk include mechanical ventilation, prolonged immobilization, persistent inflammation, concurrent infection, and dysfunction of the coagulation system and endothelium. The presence of central venous catheters, along with the use of sedatives, paralytics, and vasopressors, further increases the likelihood of DVT [2]. Despite this, ICU populations are often underrepresented in many of the studies that form the basis of current clinical guidelines.

Post-mortem studies have shown that only about one-third of PE cases in the ICU are diagnosed [1,3,4]. In a study of mechanically ventilated patients who underwent CT scans for various medical reasons, applying a standardized imaging protocol revealed PE in 18.7% of patients, with 60% of these cases being clinically silent [1]. This is particularly concerning given the high mortality associated with PE, especially when complicated by right ventricular failure or shock [5]. Untreated PE has a mortality rate of approximately 30%, but with anticoagulant therapy, this can be reduced to 2–8% [1]. The underdiagnosis of PE in the ICU is largely due to its non-specific symptoms and signs, which can be further masked by the critical condition of the patients [6]. For instance, weaning failure and persistent fever, while not commonly associated with PE, may be the only clues that prompt physicians to explore the possibility of thromboembolic disease [7,8].

This review examines the prevention, diagnosis, and management of PE from the perspective of physicians treating critically ill patients. Specifically, we will address the unique challenges of PE prevention, diagnosis, risk stratification, and management in critically ill patients, utilizing the available monitoring and diagnostic tools in the ICU setting.

## 2. Thromboembolic Prophylaxis

Even though the effect of heparin on the coagulation mechanism has been known since the 1930s, the true significance of thromboprophylaxis for preventing morbidity and mortality from PE was not realized until the 1960s, initially in postoperative patients [9,10,11,12]. In the 1980s, the discovery of low-molecular-weight heparins (LMWHs), which have a favorable pharmacokinetic profile and fewer complications, particularly a lower risk of heparin-induced thrombocytopenia (HIT), made thromboprophylaxis a standard of care, significantly improving patient outcomes [9,13]. ICU patients are at an increased risk for thromboembolic disease due to prolonged bed rest and the high prevalence of inflammation associated with severe illness [1,14,15,16,17,18,19,20].

Pharmacologic options for thromboprophylaxis in critically ill patients include both unfractionated heparin (UFH) and LMWHs/fondaparinux. In recent years, there has been a shift toward the use of LMWHs [21,22,23,24]. The PROTECT study demonstrated that, in critically ill patients, the administration of LMWH compared to UFH resulted in lower rates of PE without increasing the rates of bleeding complications and related deaths [25]. Table 1 shows the agents used in various studies for DVT and PE prophylaxis in critically ill patients and their dosages [11,26,27,28,29,30,31].

The importance of pharmaceutical thromboprophylaxis in promoting positive patient outcomes and preventing thromboembolism is well established. However, certain patient groups have absolute contraindications to anticoagulation [32,33,34]. These contraindications include active bleeding or a high risk of bleeding, particularly intracranial hemorrhage, such as that seen in a recent traumatic brain injury or hemorrhagic stroke, where anticoagulation decisions must be made in consultation with a neurosurgeon or neurologist [35,36,37]. Severe thrombocytopenia is another contraindication, though the precise platelet count threshold remains unclear. Current recommendations suggest that thromboprophylaxis be administered when the platelet count exceeds 50,000, although the supporting data are limited [11]. In cases where the platelet count is between 20,000 and 50,000, prophylactic anticoagulation may be considered with caution and under close monitoring, with decisions individualized to the patient’s specific situation [38,39,40].

Other contraindications include an allergy or history of allergy to heparin, necessitating the use of alternative prophylactic measures, and the presence of serious coagulation disorders [41,42,43]. For patients who cannot receive immediate pharmacological prophylaxis, mechanical prophylaxis, such as using intermittent pneumatic compression or graduated compression stockings, remains an option [44,45,46,47], with more evidence in support of intermittent pneumatic compression [48,49].

A subset of patients receiving heparin (UFH or LMWH) develop a clinical syndrome characterized by thrombocytopenia and life-threatening thrombosis, namely, heparin-induced thrombocytopenia (HIT), which is a complication of heparin therapy caused by antibodies against platelet factor 4 and heparin [1]. The clinical diagnosis is based on the presence of thrombocytopenia and/or thrombosis in temporal association with heparin therapy, while other causes of thrombocytopenia need to be excluded [50]. This is particularly challenging in ICU patients, who often have other potential causes of thrombocytopenia, such as sepsis, drugs, bleeding, and surgeries [51,52]. In most patients, thrombotic complications occur concurrently with thrombocytopenia and can affect any vessel, although venous thromboses predominate, especially at sites of vascular injury from catheters.

The diagnosis of HIT cannot be confirmed without laboratory detection of anti-PF4/heparin antibodies [53]. Treatment is initiated upon suspicion of HIT and includes discontinuation of all heparin sources and administration of an alternative parenteral thromboprophylaxis agent. Direct thrombin inhibitors, such as bivalirudin and argatroban, are preferred in critically ill patients. Other options include danaparoid and fondaparinux [49]. The risk of HIT is lower with LMWHs compared to that with UFH, and even lower, but not zero, with the use of fondaparinux [54].

Despite guidelines recommending the administration of thromboprophylaxis, a significant percentage of ICU patients do not receive it timely, either because there are delays in its prescription or because it is not administered as prescribed [55]. Such delays may be related to the perceived bleeding risks. A strategy used to reduce the bleeding risks related to thromboembolic prophylaxis in critically ill patients is to monitor anti-Xa activity. This approach is particularly suitable for ICU patients who often present with organ failure, including renal failure, which affects the pharmacokinetics of heparin. Monitoring anti-Xa activity can prevent both overdosing and under-dosing of LMWH, thus protecting patients from both bleeding and thromboembolic risks [56,57]. Desired anti-Xa targets for thromboprophylaxis when twice daily enoxaparin is used are reported to be 0.2 to 0.4 IU/mL, measured 3–4 h after the last dosing and following at least 3 consecutive doses [57]; however, clinicians are advised to consult local hematology and laboratory protocols because these thresholds are assay and agent dependent. It is, therefore, crucial to have therapeutic hospital protocols that specify the dosage, medicinal substance, and method of monitoring the antithrombotic effect when this strategy is applied.

## 3. Clinical Suspicion, Diagnosis, and Risk Stratification

Diagnosing PE in the ICU largely depends on the clinical suspicion of the physicians managing each patient, as the signs and symptoms of PE are often non-specific [58,59,60]. It is not uncommon for PE to be discovered incidentally during a CT scan performed for another reason [61]. The symptoms and signs that typically characterize PE in the emergency setting, such as chest pain, dyspnea, and hemoptysis, may go unnoticed due to sedation and mechanical respiratory support or may be attributed to other causes, such as mechanical injury to the tracheal mucosa or pre-existing coagulation and platelet disorders [62]. The most common triggers of PE suspicion in the ICU are persistent, unexplained hypoxemia, persistent, unexplained tachycardia, and unexplained hypotension [63,64].

An important step in diagnosing PE is evaluating the patient’s likelihood of having the condition based on clinical criteria. For this purpose, the Geneva and Wells rules are commonly used. According to the simplified Geneva clinical prediction rule, a score of 0–2 suggests PE is unlikely (see Table 2), while a score of ≥3 indicates PE is likely. In the simplified Wells prediction rule, the probability of PE is categorized as low (0–1 points), intermediate (2–6 points), or high (≥6 points). Both scales, along with their simplified versions, have been validated in large populations and are useful for assessing patients, though they are not specifically designed for critically ill populations (Table 2) [61]. As one can notice, several parameters can be affected by critical illness.

### 3.1. The Role of Continuous Hemodynamic Monitoring

Most patients admitted to the ICU are closely monitored using both invasive and non-invasive hemodynamic and respiratory techniques. Respiratory monitoring indicators that could prompt concern for PE include new or persistent hypoxemia in a patient with a normal chest X-ray, tachypnea, and hypocapnia—or hypercapnia in mechanically ventilated patients. Symptoms such as chest pain and dyspnea, which are often associated with PE, may go unreported in critically ill patients due to sedation or analgesics being administered for other reasons. Hemodynamic monitoring signs that suggest PE include electrocardiogram changes indicative of right ventricular strain, such as the “S1Q3T3” pattern (McGinn–White sign), which includes a large S-wave in lead I, a Q-wave in lead III, and an inverted T-wave in lead III. Other possible signs are T-wave inversion in leads V1–V4, a QR pattern in lead V1, or a new right bundle branch block, though these are typically seen in more severe cases. In the ICU, the most frequently encountered signs that raise clinical suspicion for PE include new-onset sinus tachycardia, new-onset atrial fibrillation, and non-specific ST interval abnormalities [62].

In addition to standard hemodynamic monitoring, many patients, especially those who show hemodynamic instability, are also monitored with invasive devices, such as pulmonary artery catheters, transpulmonary thermodilution catheters (PiCCO), or the less invasive method of pulse-wave analysis [65,66,67]. These methods do not have specific diagnostic values for PE, but their indications can be affected. Pulmonary artery catheters, although used less frequently [68,69], may show an increase in pulmonary vascular resistance. An elevated central venous pressure (CVP) greater than 20 mmHg, an inverse pressure gradient (CVP > pulmonary artery wedge pressure), and a low cardiac index (<2 L/min/m^2^), stroke volume index (<30 mL/m^2^), and mixed-venous oxygen saturation (SvO2 < 55%) are findings attributed to right ventricular dysfunction [70]. In cases of severe, massive PE, there may be a drop in blood pressure and cardiac output, a decrease in stroke volume, an increase in central venous pressure, and an increase in peripheral vascular resistance, while the wedge pressure is not expected to show significant changes. If the embolism affects the vessel where the catheter is located, it may be impossible to take measurements [71]. If PiCCO is applied in a patient with PE, then the underestimation of the extravascular lung water value has been reported [72]. The increasingly used, mainly in perioperative patients, pulse-wave analysis techniques [73] may show a drop in the cardiac index, a drop in blood pressure, a decrease in the oxygen saturation of mixed venous blood (SVO_2_), and an increase in the efficiency index of fluid responsiveness (stroke volume variation (SVV)) [74]. These findings from continuous hemodynamic monitoring may prompt the clinician to further investigate the patient.

### 3.2. The Role of Imaging and Other Diagnostic Tests

Pulmonary CT angiography (CTPA) with a PE protocol is the test of choice for diagnosing PE [2]. CTPA has replaced pulmonary angiography, the historical standard [75]. Lung scintigraphy, which is applied outside the ICU, particularly in otherwise healthy, low-risk, ambulatory cases with normal X-rays, is not commonly used in critically ill patients due to technical difficulties, longer examination times, radiation exposure to ICU staff, and high rates of inconclusive examinations, since most critically ill patients also have abnormal chest X-ray examinations [76]. CTPA is available in most hospitals, and has a sensitivity and specificity of 83% and 96%, respectively, for PE diagnosis [77]. Its disadvantages include radiation exposure, potential adverse reactions to the contrast agent, and—especially in critically ill patients—the difficulties and risks associated with patient transportation to the radiology department [78,79]. Another parameter that should not be underestimated is the risk of contrast-induced nephropathy. Although high rates of nephropathy have been reported for CTPA [80,81], this risk should not preclude clinicians from examining critically ill patients when PE is suspected.

The use of ultrasound has greatly expanded in the ICU, and training in it has become essential for the physicians in the ICU [82]. This expansion is due to the technological advancements of the machines and the fundamental characteristics of ultrasound, which do not expose the patient and staff to radiation and can be performed at the bedside [83,84]. Echocardiographic findings associated with PE are the presence of a mobile clot in the right heart chambers and the signs of right ventricular dilation and dysfunction. These include right ventricular dilation, McConnell sign, flattening of the interventricular septum, dilation of the inferior vena cava with reduced inspiratory collapse, the “60/60” sign, the presence of a mid-systolic notch, a mobile clot in the right heart chambers, decreased tricuspid annular plane systolic excursion (TAPSE) measured with M-Mode (<16 mm), and decreased peak systolic (S′) velocity of the tricuspid annulus (<9.5 cm/s) [85,86]. Many of these signs are not specific and may be seen in other respiratory and cardiac diseases [87]. The most specific signs for the diagnosis of PE are the “60/60” sign (i.e., a pulmonary ejection acceleration time < 60 ms with a peak systolic tricuspid valve gradient < 60 mmHg) and the McConnell sign (akinesia of the mid-free right ventricular wall with preserved apical contractility) [86,88]. Their presence in a patient with shock and high clinical suspicion for PE supports the diagnosis of PE and justifies the administration of thrombolytic therapy, while their absence in the same setting largely excludes PE as the reason for hemodynamic compromise. However, they are not often reported in ICU patients and are only detected in <20% of unselected PE cases; therefore, their absence also does not exclude the possibility of PE [89].

Since PE usually originates from emboli in the lower extremities (i.e., deep vein thrombosis), performing compression ultrasound to identify such thrombi can also contribute to the diagnosis [90]. For this purpose, a two-point compression ultrasound is sufficient and should be performed on both extremities at the inguinal and popliteal fossae bilaterally. In patients with high clinical suspicion, this method is sufficient for diagnosis and justifies the initiation of therapeutic measures [91].

Regarding lung ultrasound, it is not expected to show abnormal findings in PE, and in such patients, A-lines are typically seen. Other non-specific signs that may be found include wedge-shaped infarcts and pleural effusions. It should be noted that a similar appearance to the wedge-shaped infarct of pulmonary embolism can also be seen in COVID-19 disease. However, pulmonary infarcts in COVID-19 have a different pathophysiology, resulting from in situ thrombosis in the pulmonary parenchyma rather than embolism [92]. Lung ultrasound can also identify other causes of respiratory distress, such as pneumothorax, pneumonia, and adult respiratory distress syndrome (ARDS) [93].

One protocol that specifically addresses patients with suspected PE and has been proposed in recent years is the triple-POCUS (Point-of-Care Ultrasound) protocol [94]. This was first proposed by Nazerian et al. in 2014 [95] and later by Koenig et al. [96], and includes a combination of cardiac ultrasound, lung ultrasound, and lower extremity compression ultrasound [95,96]. It can be particularly useful in reducing the number of needed CTPAs in cases where the triple-POCUS protocol is negative and lung ultrasound reveals an alternative diagnosis in the emergency department setting, but there is no supportive evidence of its application in the ICU [95,96]. It is also useful to remember that a DVT diagnosis with lower limb ultrasound is a particularly specific finding for PE in patients with high clinical suspicion, even more specific than right heart dilation [91].

The blood test most associated with the diagnosis of PE is the D-dimer test. D-dimers rise in plasma when thrombosis is present due to the activation of coagulation and fibrinolysis [97], and they have a very high negative predictive value for the exclusion of PE in patients with a low pre-test probability, such as those with a low Wells score (see Table 2) [97]. D-dimers can be elevated in cases of concomitant liver disease, inflammation, malignancy, recent trauma and surgical operations, and older age, which are common in critically ill patients [98]. Therefore, measuring D-dimers is not as helpful in diagnosing PE in ICU patients, and is not recommended in this setting.

### 3.3. Risk Stratification

Risk stratification, as outlined in Table 3, is critical in guiding patient management by linking diagnosis to treatment. However, in the ICU, applying this stratification can be challenging, as many patients in the ICU already have respiratory insufficiency, hemodynamic instability, and organ dysfunction due to factors unrelated to PE. Risk stratification is typically divided into three levels: high, intermediate, and low risk. The intermediate-risk group is further subdivided into intermediate–high and intermediate–low risk. This classification is based on four main criteria: clinical signs of severity, the presence or absence of hemodynamic instability, imaging and ultrasound evidence of right ventricular strain, and biochemical markers of myocardial injury.

Clinical criteria are summarized in the Pulmonary Embolism Severity Index (PESI) and its simplified version (sPESI), which is shown in Table 4. For the simplified Pulmonary Embolism Severity Index, 0 points is related to a low 30-day mortality risk of 1.0%, and ≥1 point(s) is related to an increased 30-day mortality risk of 10.9% [1]. Key factors, such as heart rate, systolic blood pressure, and oxygen saturation, can be influenced by various conditions during critical illness and mechanical ventilation, making it essential to monitor patient trends over time for accurate risk stratification. Hemodynamic instability in the setting of risk stratification is defined by the presence of cardiac arrest, systolic blood pressure below 90 mmHg, the need for vasopressors to maintain systolic BP at or above 90 mmHg despite adequate fluid resuscitation, or a systolic BP drop of 40 mmHg for more than 15 min. It may be difficult to assess due to coexisting conditions, such as arrhythmias, hypovolemia, sedative use, or sepsis, and further obscured by prior vasopressor administration [102].

Signs on CTPA indicating right ventricular dysfunction include possible dilation of the right heart chambers and contrast reflux into the inferior vena cava [103]. The echocardiographic signs assisting risk stratification of early mortality are those reported earlier for the diagnosis of PE [85,86]. Since most of these signs are not specific to PE, baseline echocardiographic assessments may be particularly useful because they allow clinicians to track changes over time and assess the impact of new events, such as PE [62]. A novel echocardiographic sign, increasingly used for the assessment of the severity of pulmonary hypertension in PE, is the TAPSE/systolic pulmonary artery pressure ratio (TAPSE/sPAP) [104,105]. The TAPSE/sPAP ratio represents a non-invasive measure of the coupling of the right ventricle to the pulmonary artery, and for this reason, is a useful tool for risk stratification [104,105].

The increased serum levels of biochemical markers used for PE risk stratification may also be challenging to interpret. Rising serum levels of cardiac troponins can be attributed to demand ischemia occurring in sepsis, hypotension, and rapid atrial fibrillation, or to impaired troponin clearance due to acute kidney injury [106]. There are no specific thresholds to definitively rule out pulmonary embolism as the cause of elevated troponin levels when PE is suspected [107]. Since all biomarkers have similar limitations, clinicians need to understand their physiological significance and account for the impact of co-morbidities when interpreting elevated values. Other biomarkers specifically related to risk stratification in PE are as follows: (a) myocardial damage biomarkers, which, as well as the cardiac troponins, also include heart-type fatty acid-binding protein (H-FABP) [108,109], (b) biomarkers of right ventricular strain, such as brain natriuretic peptide (BNP) or N-terminal pro-b-type natriuretic peptide (NT-proBNP) [110,111], and (c) other laboratory biomarkers, such as lactate [112], neutrophil gelatinase-associated lipocalin (NGAL) [113], cystatin C [113], serum Na [114], copeptin [115], creatinine, and the estimated glomerular filtration rate (eGFR; Table 5) [113].

## 4. Management of Acute Pulmonary Embolism in the Intensive Care Unit

In any diagnosed case of PE in the ICU, the clinician has to support ventilation and hemodynamics alongside specific PE treatments. Hypoxemia should be corrected with oxygen administration by nasal cannula, face mask, or high-flow nasal cannula in more severe cases. Positive ventilatory pressures may increase the afterload of the right ventricle [62]. If intubation cannot be avoided, such as in cases of shock or cardiac arrest, then all care should be taken not to exacerbate hypotension during the intubation procedure. Strategies that can be applied to this aim include adequate peri-procedural vasopressor support, using ketamine or etomidate, and even considering awake intubation if there is relevant expertise [116]. In patients where positive-pressure ventilation is warranted, every attempt should be made to keep ventilation pressures low (both plateau airway pressure and positive end-expiratory pressure) and avoid hypercapnia, which can further compromise the function of the right ventricle [62,117].

**Figure 1:**
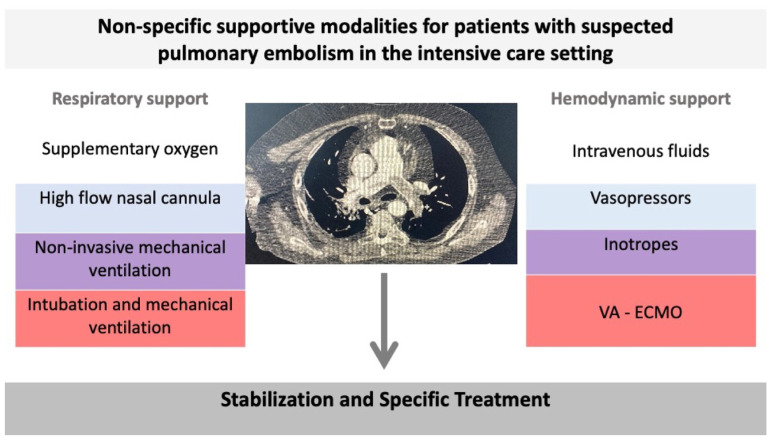
There are different non-specific modalities that can be used in the intensive care setting to support patients with pulmonary embolism with respiratory and hemodynamic compromise.

Regarding hemodynamic support, intravenous fluids, vasopressors such as noradrenaline and vasopressin, and inotropes such as dobutamine may be necessary, depending on the right ventricular status usually assessed by point-of-care cardiac echo [118]. The target here is to optimize the cardiac preload, but aggressive fluid administration is avoided because it can increase right ventricular distension and further compromise the cardiac output [62]. Noradrenaline is currently considered the catecholamine of choice for maintaining arterial blood pressure, and dobutamine in doses < 10 μg/kg/min is the inotrope of choice for the support of right ventricular contractility. Figure 1 summarizes the non-specific modalities that can be applied in this setting. Guidelines emphasize that VA-ECMO should be considered in combination with a reperfusion strategy in patients with PE and refractory circulatory collapse or cardiac arrest [88,119]. VA-ECMO can also offer salvage therapy for patients unresponsive to treatment or with contraindications to thrombolysis [120]. However, there are no randomized controlled trials on the efficacy and safety of VA-ECMO in high-risk PE [120,121].

Specific management for PE involves therapeutic anticoagulation for low- and intermediate-risk patients and lung reperfusion treatment for high-risk patients [11,88]. The intermediate–high-risk patients require close monitoring, and immediate reperfusion treatment if there is clinical deterioration, such as hemodynamic instability [122]. Reperfusion strategies include the following: (a) intravenous systemic thrombolysis, (b) surgical embolectomy, and (c) catheter-directed thrombus removal [123]. All these strategies need to be followed by therapeutic anticoagulation [88]. The successful management of PE in critically ill patients requires informed and prompt decision-making, and complex cases often require collaboration with specialists in respiratory medicine, cardiology, surgery, and interventional radiology [124,125]. To address this aspect of PE care, establishing pulmonary embolism response teams (PERT) has been suggested and implemented in several hospitals. It has been shown that implementing PERTs is associated with higher rates of advanced therapies, such as catheter-directed embolectomy and VA-ECMO, being applied. Further studies are needed, however, to prove the role of PERTs in improving outcomes [126,127].

### 4.1. Therapeutic Anticoagulation

Therapeutic anticoagulation is essential for most ICU patients with PE who are not assessed as high risk and are hemodynamically stable or, if unstable, are not considered so due to the PE. In ICU patients, parenteral anticoagulants are used, and they are shown in Table 6 [88]. Contraindications to therapeutic anticoagulation, such as an increased risk of bleeding due to recent surgery or intracranial pathology, should be evaluated with caution, as delaying anticoagulation may increase the risk of recurrent PE [128]. Special characteristics of critically ill patients, such as potentially impaired absorption following subcutaneous administration, decreased clearance due to renal impairment, altered drug distribution following fluid resuscitation and vasopressor use, as well as the need for unplanned invasive procedures, may increase the risk of bleeding complications with LMWH [2]. A strategy that can be implemented to reduce the bleeding risk and optimize management is to monitor therapeutic anticoagulation by assessing anti-Xa activity 4 h after administration of LMWHs. Anti-Xa peak-level targets for the therapeutic, twice daily administration of LMWHs are 0.5–1.2 IU/mL; however, it is advised that clinicians follow their laboratory and hospital guidelines for the specific agent used, including fondaparinux, as well as for the appropriate sampling times [129].

The use of UFH may be considered in patients at higher risk of hemorrhage, as it can be immediately discontinued and rapidly reversed. UFH is also a choice for patients with hemodynamic instability or risk of imminent hemodynamic decompensation, in whom reperfusion treatment will likely be necessary [130]. Few patients, particularly when a concomitant inflammatory disease is present, require large doses of UFH to achieve a therapeutic-activated partial thromboplastin time. Despite aPTT being suboptimal, patients may have adequate heparin levels upon protamine titration. Monitoring the anti-factor Xa assay in this situation may result in less escalation of the UFH dose [131].

### 4.2. Inferior Vena Cava Filters

Placement of inferior vena cava filters (IVCFs) aims primarily to prevent peripheral thrombi from reaching the pulmonary circulation [88]. Reasons for their applications include diagnosed deep vein thrombosis in the lower limbs with an absolute contraindication to therapeutic anticoagulation, and recurrent PE in patients on adequate therapeutic anticoagulation. If a delay in anticoagulation initiation is necessary, the use of an IVCF can be considered [128]; however, routine use of IVCFs for PE prevention is not currently advised [132]. Common complications of IVCFs are periprocedural, such as penetration of the venous wall [133]. Other complications include filter embolization and thrombosis [134]. IVCFs should be removed as soon as they are no longer needed, such as when anticoagulation can be safely initiated. Filter infection is a concern in critically ill patients with or at risk of sepsis, which may require filter removal [2].

### 4.3. Reperfusion Therapies

For patients with high-risk PE, and some patients stratified in the intermediate–high-risk group, reperfusion therapy with systemic thrombolysis is indicated [88,135]. Recombinant tissue plasminogen activator (rtPA), streptokinase, and urokinase are used for this purpose, with dosages provided in Table 7. While systemic thrombolysis carries a significant risk of major bleeding, including intracranial hemorrhage, delays or reluctance in its administration can result in poorer outcomes in high-risk PE [136]. Absolute contraindications include major surgery or trauma in the preceding three weeks, active bleeding or bleeding diathesis, a history of hemorrhagic stroke, ischemic stroke in the past six months, and known central nervous system neoplasm. Relative contraindications include non-compressible puncture sites, traumatic resuscitation, active peptic ulcer, refractory hypertension with systolic blood pressure > 180 mmHg, transient ischemic attack in the past six months, prior oral anticoagulation, pregnancy, the first post-partum week, advanced liver disease, and infective endocarditis [88,135]. For the intermediate–high-risk cases, close observation is indicated, because this group does not benefit as much from thrombolysis, as bleeding complications do not meet the improvement of hemodynamic compromise. Although, the guidelines do not support thrombolytic therapy for these patients, while catheter-directed techniques are increasingly reported in this setting and show promising results [122,137].

When systemic thrombolysis is contraindicated, alternative revascularization options are surgical embolectomy and catheter-directed treatments [88]. Surgical embolectomy is considered when thrombolysis fails or is contraindicated and is performed with cardiopulmonary bypass. It has shown promising results also in resource-limited settings, although there are no randomized trial data to compare it with systemic thrombolysis. The procedure carries risks and depends on local resources and expertise [123,138]. Catheter-based approaches, including catheter-directed thrombolysis and catheter-based embolectomy (or a combination of both), have gained interest over the past decade and are increasingly used in hemodynamically unstable patients, more frequently than surgical embolectomy [139]. However, their effectiveness compared to systemic thrombolysis remains unclear, as adequately powered studies in high-risk PE patients are currently unavailable [123,135].

## 5. Conclusions

The guidelines for the diagnosis, prevention, and management of PE provide the basis for clinicians caring for susceptible patients. Familiarity with these guidelines is essential. Critically ill populations present complex cases, necessitating a personalized approach due to the unique challenges and pathophysiological derangements. High risks of bleeding, low platelet counts, impaired renal function, and altered volumes of distribution can affect anticoagulation strategies. Close observation, with or without anticoagulation monitoring, can help maximize benefits and minimize risks. Diagnosing PE in critically ill, mechanically ventilated patients is more challenging because the physical signs and symptoms indicative of PE may be absent or attributable to other common conditions in critically ill populations, such as sepsis, delirium, acute coronary syndromes, ventilator dyssynchrony, hypovolemia, or shock of various etiologies. Treatment of PE may also be complicated by contraindications to therapeutic anticoagulation and thrombolysis. In such cases, advanced measures, such as mechanical thrombectomy, may be required, especially in perioperative, trauma, or patients with primary neural injury. Randomized controlled trials often do not include critically ill patients, making prospective observational data and case series valuable and informative for clinicians managing these complex cases.

## Figures and Tables

**Table 1:** Medications used for pharmacologic thromboprophylaxis in the intensive care unit.

Agent	Dose *
Unfractionated heparin	5000 IU × 2 SC
Dalteparin	5000 IU × 1 SC
Enoxaparin	40 mg × 1 SC
Bemiparin	3500 IU × 1 SC
Nadroparin	2850 IU × 1 SC
Tinzaparin	4500 IU × 1 SC
Reviparin	1431 IU × 1 SC
Fondaparinux	2500 IU × 1 SC

* Higher “intermediate doses” and modifications according to thromboembolic risk have also been described. Twice daily dosage and monitoring of anti-Xa activity is advisable for high-risk groups, such as trauma patients. See the text for references. IV, intravenous route of administration; SC, subcutaneous route of administration. IU, international units.

**Table 2:** The simplified Geneva and Wells clinical prediction rules and their usefulness in the ICU setting.

The Simplified Geneva Clinical Prediction Rule	Points	Useful in ICU
History of PE/DVT	1	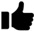
HR 75–94 b.p.m.	1	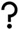
HR ≥ 95 b.p.m.	2	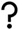
Fracture/surgery within previous 30 d.	1	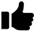
Hemoptysis	1	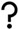
Active malignancy	1	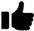
Unilateral leg pain	1	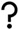
Unilateral leg edema and pain on deep venous palpation	1	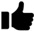
Age > 65 years	1	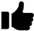
**The Simplified Wells Prediction Rule**		
Signs and symptoms of DVT	3	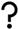
Alternate diagnosis less likely than PE	3	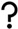
HR > 100 b.p.m.	1.5	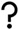
Bed-bound or surgery in the previous 28 d.	1.5	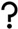
History of DVT/PE	1.5	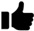
Hemoptysis	1	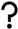
Active malignancy	1	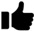

PE, pulmonary embolism; DVT, deep vein thrombosis; HR, heart; d., days; ICU, intensive care unit. Thumbs-up symbols signify that this parameter can be safely assessed in most critically ill patients. Question marks signify that the parameter is often affected by critical illness other than pulmonary embolism. Monitoring trends can be useful for the evaluation of the patient.

**Table 3:** Pulmonary embolism stratification of the risk of in-hospital or 30-day mortality and challenges for its implementation in the critically ill.

Risk of Early Mortality	Hemodynamic Instability	sPESI ≥ 1	Right Ventricular Dysfunction	Cardiac Troponin Elevation
High	+	+	+	+
Intermediate–high	-	+	+	+
Intermediate–low	-	+	+/-	+/-
Low	-	-	-	-
Challenges in critically ill patients	>50% on vasopressors [99]	>25% have non ARDS hypoxemia [100]	Can be caused by concomittant heart/lung disease, MV, and ARDS [70]	Reported rate > 40% in critically ill populations [101]

PESI, Pulmonary Embolism Severity Index; sPESI, simplified Pulmonary Embolism Severity Index (see also Table 4); MV, mechanical ventilation; ARDS, adult respiratory distress syndrome. +, present; -, absent.

**Table 4:** The simplified Pulmonary Embolism Severity Index and its usefulness in the ICU setting.

The Simplified Pulmonary Embolism Severity Index		
>80 years	1	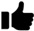
Active malignancy	1	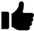
Chronic heart failure	1	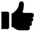
Chronic lung disease	1	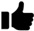
HR ≥ 110 b.p.m.	1	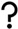
SBP < 100 mmHg	1	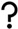
O_2_ Sat < 90%	1	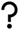

HR, heart rate; ICU, intensive care unit; SBP, systolic blood pressure; O_2_ Sat, arterial oxyhemoglobin saturation. Thumbs-up symbols signify that this parameter can be safely assessed in most critically ill patients. Question marks signify that the parameter is often affected by critical illness other than pulmonary embolism. Monitoring trends can be useful for the evaluation of the patient.

**Table 5:** Blood biomarkers related to the diagnosis and risk stratification of pulmonary embolism.

Biomarker	Reference Values	Cut-off Values for Risk Stratification	Physiological Role	Comments
High-sensitivity troponin T	<14 pg/mL *	≥14 pg/mL if <75 y.o. ≥45 pg/mL if ≥ 75 y.o.	Marker of myocardial injury	High NPV for acute PE; associated with outcomes and mortality
H-FABP	1.7 ± 0.9 ng/mL ^$^	≥6 ng/mL	Marker of myocardial injury	Associated with outcomes and mortality
BNP NT-proBNP	BNP < 100 pg/mL NT-proBNP < 125 pg/mL if <75 y.o. < 450 pg/mL if >75 y.o.	≥600 pg/mL	Index of right ventricular strain	Associated with outcomes and mortality
Serum lactate	<2 mmol/L	≥2 mmol/L	Balance between tissue oxygen supply and demand	Predictive of PE-related complications
eGFR	90 to 120 mL/min/1.73 m^2^	≤60 mL/min/1.73 m^2^	Index of renal function	Associated with 30-day mortality
NGAL	50–149 ng/mL	>75 ng/ml	Index of renal injury	Associated with 30-day mortality
Cystatin C	500–1000 ng/ml	>1900 ng/ml	Index of renal injury	Associated with 30-day mortality
Serum Na	135–145 mEq/L	<135 meq/L	Index of total body water	Predictive of in-hospital mortality
Copeptin	1–13.8 pmol/L	≥4 pmol/L	Marker of endogenous stress	Increased risk of adverse outcome

* Normal < 14 pg/mL, borderline 14–52 pg/mL, elevated > 52 pg/mL. ^$^ For subjects aged 41–69 years old, the 99th centile is 5.6 ng/mL. y.o., Years old; NPV, negative predictive value; H-FABP, heart-type fatty acid-binding protein; BNP, brain natriuretic peptide; NT-proBNP, N-terminal pro-B-type natriuretic peptide; eGFR, estimated glomerular filtration rate; NGAL, neutrophil gelatinase-associated lipocalin.

**Table 6:** Medications used for therapeutic anticoagulation for pulmonary embolism in the intensive care unit.

Agent	Dose ^a^
Unfractionated heparin	80 IU/kg IV bolus, followed by continuous IV infusion of 18 units/kg/h IV
Dalteparin	100 IU/kg × 2 SC
	200 IU/kg × 1 SC
Enoxaparin	1.0 mg/kg × 2 SC
	1.5 mg/kg × 1 SC
Nadroparin	86 IU/kg × 2 SC
	171 IU/kg × 1 SC
Tinzaparin	175 IU/kg × 1 SC
Fondaparinux ^b^	BW < 50 kg: 5 mg × 1 SC
	BW 50–100 kg: 7.5 mg × 1 SC
	BW > 100 kg: 10 mg × 1 SC

^a^ Doses may need to be adjusted according to anti-Xa serum activity. The dose of continuous unfractionated heparin infusions is adjusted, targeting an activated partial thromboplastin time of 1.5–2.3 × control. ^b^ Caution is advised with the use of fondaparinux in patients with impaired creatinine clearance due to an increased risk of bleeding. If the use of fondaparinux is necessary, e.g., in cases of heparin-induced thrombocytopenia, when alternative agents such as bivalirudin or argatroban are not available, monitoring anti-Xa activity is advised to reduce the risk of bleeding. IV, intravenous route of administration; SC, subcutaneous route of administration. IU, international units.

**Table 7:** Medications and protocols used for systemic thrombolysis in pulmonary embolism.

Agent	Dose ^a^
rtPA	100 mg/2 h, IV. In cases of extreme hemodynamic compromise, consider 0.6 mg/kg/15 min, IV (max. 50 mg)
Streptokinase	250,000 IU/50 min, then 100,000 IU/h for 12–24 h, IV
Urokinase	4400 IU/kg/10 min, then 4400 IU/kg/h for 12–24 h, IV

^a^ rtPA use is preferred over streptokinase or urokinase extended infusions. There is insufficient evidence on the use of accelerated protocols. rtPA, recombinant tissue-type plasminogen activator.
